# Foundation models for radiology—the position of the AI for Health Imaging (AI4HI) network

**DOI:** 10.1186/s13244-025-02056-9

**Published:** 2025-08-06

**Authors:** José Guilherme de Almeida, Leonor Cerdá Alberich, Gianna Tsakou, Kostas Marias, Manolis Tsiknakis, Karim Lekadir, Luis Marti-Bonmati, Nikolaos Papanikolaou

**Affiliations:** 1 Champalimaud Foundation, Lisbon, Portugal; 2 La Fe Health Research Institute, Valencia, Spain; 3 R&D Department of Maggioli SpA, Greek Branch, Athens, Greece; 4 Hellenic Mediterranean University and Foundation for Research and Technology Hellas, Heraklion, Greece; 5 Universitat de Barcelona, Artificial Intelligence in Medicine Lab (BCN-AIM), Department of Mathematics and Computer Science, Barcelona, Spain; 6 Institució Catalana de Recerca i Estudis Avançats (ICREA), Universitat de Barcelona, Barcelona, Spain; 7 Hospital Universitario y Politécnico La Fe, Valencia, Spain; 8 Royal Marsden Hospital NHS Foundation Trust, London, England

**Keywords:** Artificial intelligence, Radiology, Foundation models, Bias

## Abstract

**Abstract:**

Foundation models are large models trained on big data which can be used for downstream tasks. In radiology, these models can potentially address several gaps in fairness and generalization, as they can be trained on massive datasets without labelled data and adapted to tasks requiring data with a small number of descriptions. This reduces one of the limiting bottlenecks in clinical model construction—data annotation—as these models can be trained through a variety of techniques that require little more than radiological images with or without their corresponding radiological reports. However, foundation models may be insufficient as they are affected—to a smaller extent when compared with traditional supervised learning approaches—by the same issues that lead to underperforming models, such as a lack of transparency/explainability, and biases. To address these issues, we advocate that the development of foundation models should not only be pursued but also accompanied by the development of a decentralized clinical validation and continuous training framework. This does not guarantee the resolution of the problems associated with foundation models, but it enables developers, clinicians and patients to know when, how and why models should be updated, creating a clinical AI ecosystem that is better capable of serving all stakeholders.

**Critical relevance statement:**

Foundation models may mitigate issues like bias and poor generalization in radiology AI, but challenges persist. We propose a decentralized, cross-institutional framework for continuous validation and training to enhance model reliability, safety, and clinical utility.

**Key Points:**

- Foundation models trained on large datasets reduce annotation burdens and improve fairness and generalization in radiology.
- Despite improvements, they still face challenges like limited transparency, explainability, and residual biases.
- A decentralized, cross-institutional framework for clinical validation and continuous training can strengthen reliability and inclusivity in clinical AI.

**Graphical Abstract:**

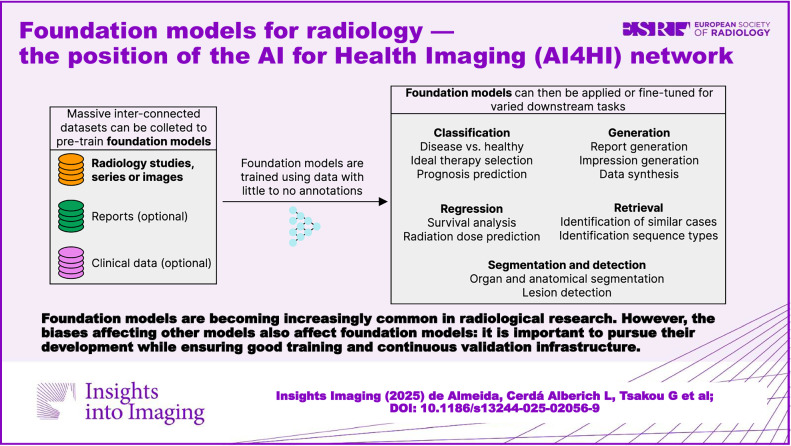

## Introduction

Medical images offer a wealth of relevant information about the patient that clinicians use for better diagnosing and treating patients. Due to their centrality in diagnosis, the number of acquired scans has steadily increased over the years [1, 2], creating a great demand for trained professionals in acquiring, interpreting and integrating results, a high workload demand which is presently hard to adequately fulfill [3–5] and leads to increasing burnout rates among radiologists [6, 7]. To make matters worse, radiographers and medical physicists are also facing a global shortage [8, 9], making this problem transversal to image scheduling, acquisition, quality control, interpretation, and reporting.

Medical imaging AI systems have recently appeared as potential aids in not only reducing the burden placed on radiologists but also improving the patient journey and healthcare. For example, sensitivity can be boosted by AI in breast cancer detection by capturing lesions missed by radiologists [10–15], in chest X-ray by nodule interpretation [16, 17], and in CT scans by intracranial hemorrhage detection [18]. Performance boosts are also observed in prostate MRI significant cancer detection by using deep learning tools while also reducing the likelihood of false positives [19, 20], in diagnosing pathological complete response in colorectal cancer using MRI radiomics [21], or in the observation and delineation of lung nodules in chest CT with convolutional neural networks [22, 23]. In radiotherapy, AI can assist in contouring (i.e., through semi-automatic or automatic segmentation of organs at risk or clinical target volumes) with consistent time reductions [24, 25]. AI can also generate synthetic CT data for MRI-only radiotherapy treatment plans using a different set of CT images obtained in real life [26].

The potential of AI in radiology, including foundation and generative models, has been discussed among experts and stakeholders from the AI for Health Imaging (AI4HI) network. The AI4HI networks connect 5 large-scale EU-funded consortia developing, validating and deploying AI for health imaging (EuCanImage, CHAIMELEON, INCISIVE, ProCAncer-I, and PRIMAGE), and incorporate professionals from both technical and clinical backgrounds alike [27–30]. Based on the experiences and results from these projects, the consensus was that while emerging AI solutions hold promise, careful attention is required regarding their robustness, generalizability, long-term performance and ethical compliance. AI-driven predictions can sometimes lead to incorrect diagnoses, which may prompt clinicians to make erroneous treatment decisions [31, 32]. These issues largely stem from clinical AI models’ failure to generalize to external datasets or under-represented populations [33, 34].

In chest X-ray classification using clinical AI models, Rudolph and others highlighted how patient positioning could impact the performance of AI systems on external datasets [35], while Kim et al showed that shifts in disease prevalence between deployment and training cohorts could cause significant performance reductions [33, 36]. Differences in performance are particularly concerning when they disproportionately affect specific demographic groups, such as certain countries, races, genders, or age groups [37, 38]. Biased models that underperform for specific groups can result in overtreatment or underdiagnosis, depending on the direction of the bias.

Furthermore, the large data requirements of traditional AI systems may cause them to underperform when diagnosing relatively rare conditions [39]. For example, a model trained to diagnose rare syndromes based on facial recognition underperformed in non-white populations [40]. Hidden stratification—when there are underlying but unobservable subsets of data with different levels of performance—can also lead to underperforming models in unforeseen circumstances [41]. These disparities not only affect patients but also have implications for healthcare providers, as declining performance may expose institutions and clinicians to legal liability for inaccurate predictions [42].

Finally, systematic and local assessments have indicated that AI systems may struggle to maintain consistent performance, both at launch and over time, hindering their viability as clinical tools [43, 44]. Additionally, differences between datasets in terms of center, acquisition protocol and scanner manufacturer can hinder model performance [33, 36]. These phenomena, which may involve shifts in the spectrum of data, acquisition software, and clinical targets, cause AI models to degrade over time if not regularly monitored and updated [45, 46]. Continuous evaluation and recalibration are essential to ensure that AI systems remain tuned and effective in clinical practice, particularly as medical conditions, imaging technologies, and patient demographics evolve. To rigorously validate AI models and account for biases, large demographically balanced datasets with annotations are needed; this can oftentimes be unrealistic due to economical, legal and ethical constraints.

## Foundation models: potential and limitations

**Foundation models** are large-scale AI models pre-trained on diverse, non-specific tasks (such as language generation, image captioning, or image reconstruction) and serve as adaptable starting points for specialized applications [47]. Some foundation models fall under the umbrella of generative AI, a subset of AI that focuses on synthesizing new content, be it text, images, video, voice, or other information modalities. Generative AI leverages these foundational architectures to produce novel outputs, while foundation models more broadly serve as a base for both generative and non-generative applications. For example, in healthcare, generative AI powered by foundation models enables tasks like synthetic data generation for rare disease research or personalized treatment simulations [48], whereas non-generative applications encompass clinical decision support systems, diagnostic assistance, and workflow optimization, which aid in improving diagnostic accuracy and clinical efficiency [49]. To assist the reader, we provide a glossary containing helpful definitions relating to foundation models used through this piece (Table 1).

**Table 1:** A short glossary containing helpful concepts when reading about and discussing foundation models

Concept	Explanation	Examples
Foundation models	Large-scale artificial intelligence models that are pre-trained on broad tasks and serve as adaptable starting points for specialized applications. Foundation models are also considered to be more data-efficient than supervised alternatives.	Large language models, vision-language models, SAM [66], MedSAM [65], nnInteractive [121]
Pre-training	Using large amounts of data to train a model that can be adapted to other tasks.	See Table 2.
Data efficiency	Data efficiency is generally described as the amount of data required to obtain a given level of performance. Typically, foundation models are more data-efficient than models that are trained from scratch when fine-tuned on downstream tasks.	
Large language models (LLMs)	Transformer-based models [51] are trained on vast amounts of text. The pre-training stage of these models focuses on using parts of sentences to predict the next or the following parts of that same sentence. This gives these models a general understanding of language. They can then be further used across a variety of tasks [122, 123] or fine-tuned to perform tasks such as text summarization [108], radiological impression generation from radiological reports [70] or used as chatbots [52]. When LLMs are not fine-tuned but are instead directly used as they are, this constitutes a form of zero-shot or few-shot learning.	The GPT family of models (starting with GPT-2 [122]), LLaMa [118], DeepSeek [124], Gemma 3 [125]
Vision language models (VLMs)	Vision language models combine the text-generation capabilities of LLMs with generic visual understanding. These models are typically composed of a “vision module” (responsible for characterizing each image as a vector), a “projector module” (responsible for aligning the image vectors with text), and a “text-generation module” (responsible for generating text based on a combination of projected image vectors and text). These models are typically trained similarly to LLMs, with images also being a part of the context for prediction.	LLaMa 3.2 [126], Gemma 3 [125]
Supervised learning	Supervised learning is a framework for training machine-learning models where a set of input data is associated with task-specific outputs, which act as prediction targets. In other words, a supervised learning model is trained to predict, from its input data, a given output category or quantity.	Training a model to predict lung pathologies from chest radiographs
Self-supervised learning	Self-supervised learning, unlike supervised learning, uses the input itself as the prediction output and requires no task-specific outputs.	Training an LLM to predict the next part of a sentence or training an image model to predict parts of the image that have been removed. See “contrastive learning” and “masked autencoder” in Table 2.
Training from scratch	Training a model “from scratch” means training a model that has not been pre-trained. In other words, when training a model from scratch, model weights are initialized randomly. This generally takes longer to optimize, as foundation models or other pre-trained models have already been pre-trained in a way that is relevant for downstream tasks.	
Fine-tuning	The process of adapting a pre-trained model to a related or novel task. This process, unlike zero-shot or few-shot learning, involves updating the model parameters (typically known as model weights).	MedSAM, a generalist model for medical image segmentation, is a fine-tuned version of SAM, a generalist model for image segmentation.
Open models	Open models are models whose model weights—at least—are made available. In other words, provided sufficient computational resources, individual users can use these models on their local machines without having to upload their data to the cloud to interact with models.	Individual users can interact with LLMs and VLMs through frameworks such as Ollama [127], based on llama.cpp [128], or HuggingFace [129].
Model weights	In deep learning, model weights refer to the parameters that transform an input into its respective output. Similar to more traditional data-driven methods such as linear regressions, model weights are inferred directly from the data.	
Few-shot learning or in-context learning	Few-shot learning, also known as in-context learning, is a process that allows models to perform tasks with a limited number of (or a few) examples. When a single example is provided, this is known as one-shot learning. We note here that the concept of few-shot learning is not unique to foundation models and is more traditionally associated with meta-learning, an area that also focuses on training models to learn from a limited set of examples.	In LLMs: providing a few examples in the prompt. In VLMs: providing a few images with their respective annotations as a part of the prompt. In more traditional scenarios: associated with training models to learn from limited examples (as in meta-learning) or with approaches such as metric-learning, where a model classifies an example based on its feature-distance to a limited number of examples.
Zero-shot learning	Generally, zero-shot learning involves querying the model to provide an answer without providing any examples. Alternatively, zero-shot learning tasks can also make use of examples (similar to few-shot learning), but for which no examples were available in the training data. However, the latter definition is quite similar to that of few-shot learning.	In VLMs: providing an image with a prompt such as “this is an image of” and allowing the model to automatically complete the rest of the prompt with the answer.

While examples are provided, these are not meant to be extensive but rather illustrative.

Foundation models improve the performance of AI in specific tasks by leveraging very large amounts of data which have, usually, not been labeled or otherwise curated. Such training datasets have advantages: not only do they better represent the variations in quality observed in real-world data, but they are also more varied in terms of populations and acquisition conditions or parameters. Importantly, such data are easier to obtain, considering that they require less effort and time to collect and make available for AI model training. By leveraging these large, readily available and uncurated collections of data, powerful foundation models can be trained.

Many foundation models are used in the form of chat interfaces based on large language models (LLMs), which are among the earlier forms of foundation models [50]. LLMs are immensely complex models (typically more than 1 billion parameters) that are usually based on the transformer architecture [51] and capable of generating text similar to that of a human. Training an LLM is typically straightforward: the process focuses on predicting what the next word or token (a part of a sentence or word) can be [51]. By significantly scaling up this process both computationally and in terms of data, these models have been shown to accurately reproduce text similar to that of a human. Foundation models are developed not only for text-based tasks but also for other modalities, such as images, or combinations of information sources, such as vision language models (VLMs), which incorporate images and text into their processing capabilities.

**Fig. 1:**
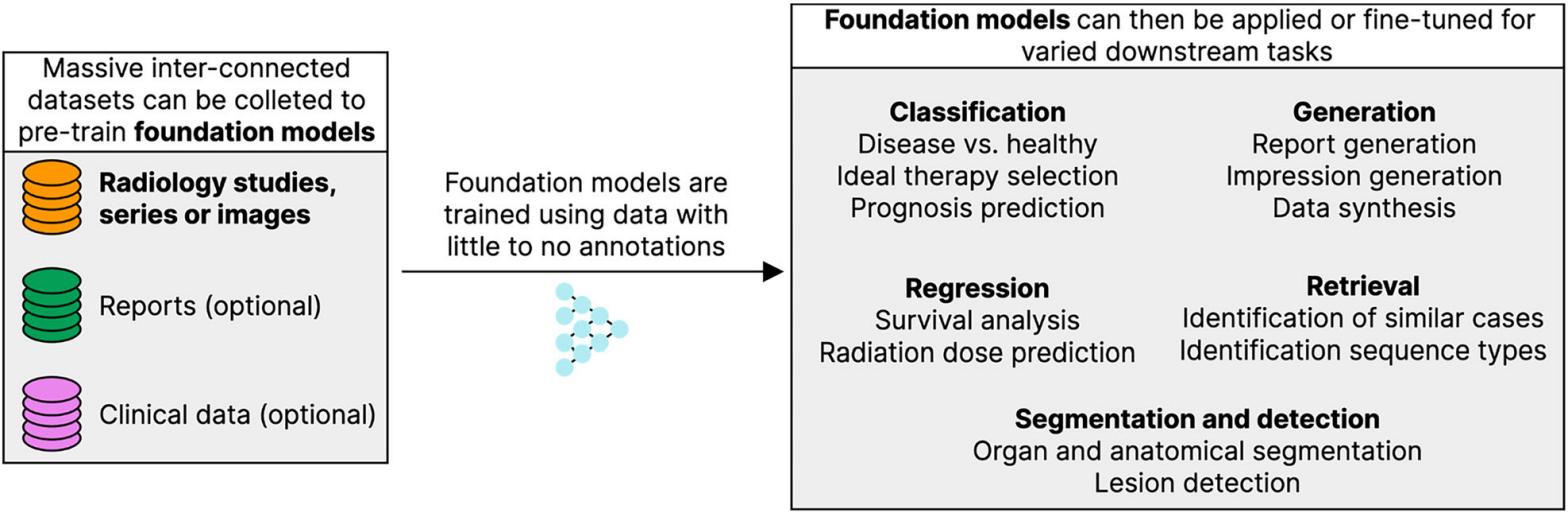
Typical foundation model workflow. Foundation models for radiological images are trained using different sources of information (radiological studies, series or images and, optionally, reports or clinical data). After training, foundation models can then be further applied or fine-tuned to a wide variety of clinically relevant downstream tasks

Building a foundation model typically involves a “pre-training” process, i.e., a process during which models are trained on vast amounts of data that may have minimal or no annotations (Table 2). This core aspect of foundation models—the existence of a pre-training stage —is crucial for their downstream performance when foundation models are adapted to perform specific tasks (Fig. 1). For instance, in chatbots, pre-training is typically followed by some form of optimization which makes them more conversational and reduces the possibility of these models producing harmful output [52]. LLMs in the medical domain may be pre-trained on very big medical text datasets like electronic health records (EHRs), clinical notes, and scientific literature. In other cases, the solution of fine-tuning general (non-medical)-purpose LLMs with high-quality, curated medical data is preferred [53] to avoid the high computational expenses linked to pre-training. In medical image analysis, pre-training is typically followed by prompting or fine-tuning general models to perform specific tasks on specific anatomies and diseases, such as medical image segmentation, cancer diagnosis, or disease prediction. When pre-training uses no labels (as is the case with LLMs), this process can also be known as self-supervised learning, since the “supervision” (typically segmentation or classification labels) are the input data themselves (in the case of LLMs, the next token or word in a sentence provides the supervision). Some more recent approaches focus on what is known as “AI agents” (the moniker provided to specialized LLMs, VLMs, and other ML models capable of interacting with one another and with computational tools, functions, or software programs) and show potential application in oncological diagnosis and research [54, 55]. Multi-agent frameworks, which coordinate multiple AI agents, have been further posited as an essential step in further advancing the collaboration of clinicians with AI systems by automatically triggering specialist AI agents in an automated or semi-automated fashion [56, 57].

**Table 2:** Summary of some pre-training strategies that can be used to train foundational models using image data

Pre-training strategy	Data requirements	Description
Caption or report generation	Image-text pairs	A model is trained to predict the most likely caption or report from a given input image.
Masked autoencoder	Images	A model is trained to predict patches that have been removed (masked) from a given input image.
Contrastive learning	Images	A model is trained to produce similar numerical representations (vectors) for the same image after distinct image transformations. Examples of such transformations include rotations, noise addition or partial image masking.
Supervised pre-training	Image-label pairs	A model is trained on a large dataset (i.e., natural image datasets such as ImageNet [130] or chest radiograph datasets such as VinDr-CXR [131] or PediCXR [132] can be used for classification, or others, such as SAROS, containing 900 fully segmented CT scans from multiple other datasets [133], or the dataset used to train the MedSAM model [65], can be used for segmentation pre-training), for which image-label pairs are available.

**Fig. 2:**
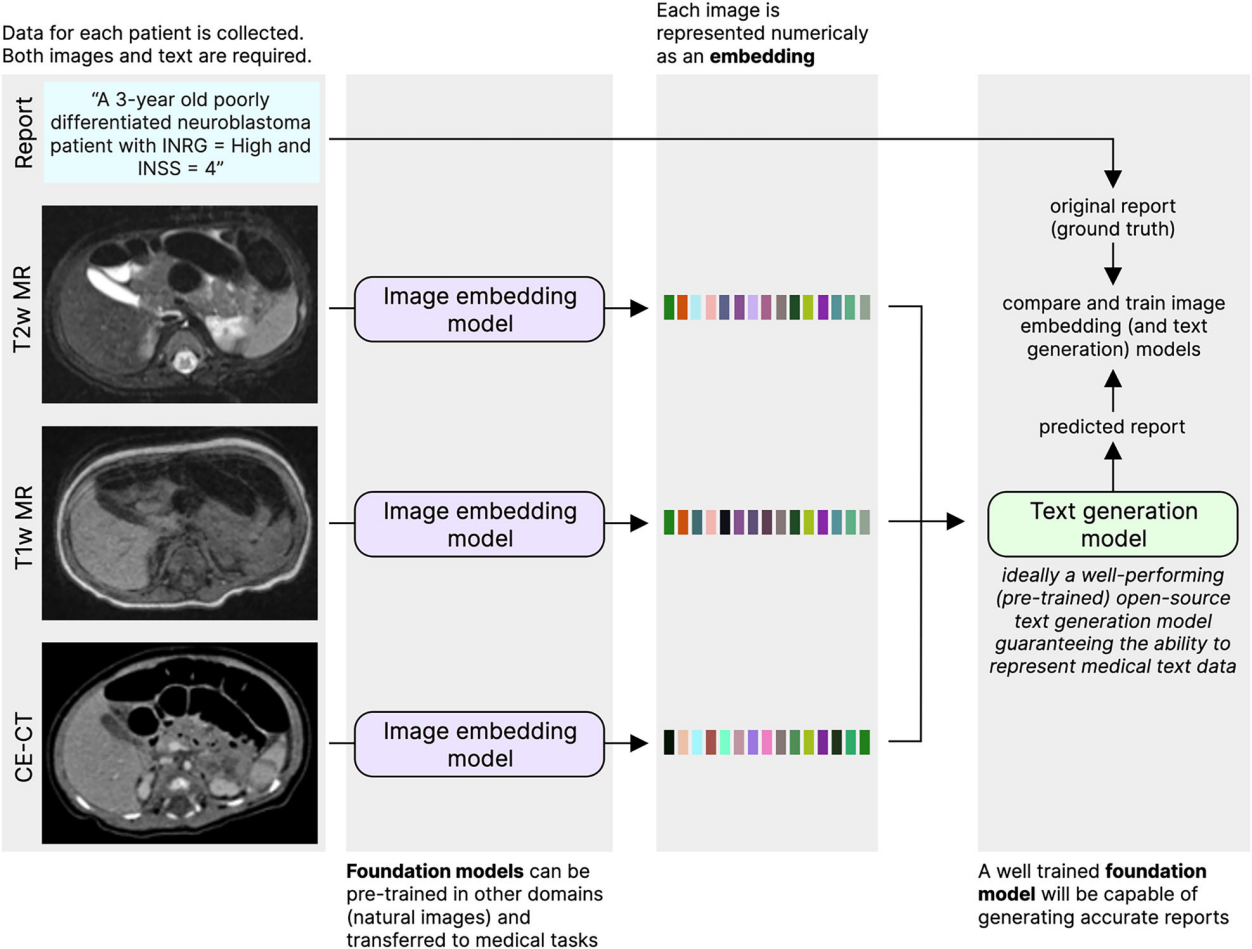
Pre-training with report generation. Input images must have paired reports. Each image is converted to a numerical embedding, which is used as input for a text generation model. The generated text is compared with the original report, and the model is trained to generate reports that closely resemble those provided as ground truths. This leads models to learn how to generate embeddings that closely relate to the reports, thus capturing relevant diagnostic information

**Fig. 3:**
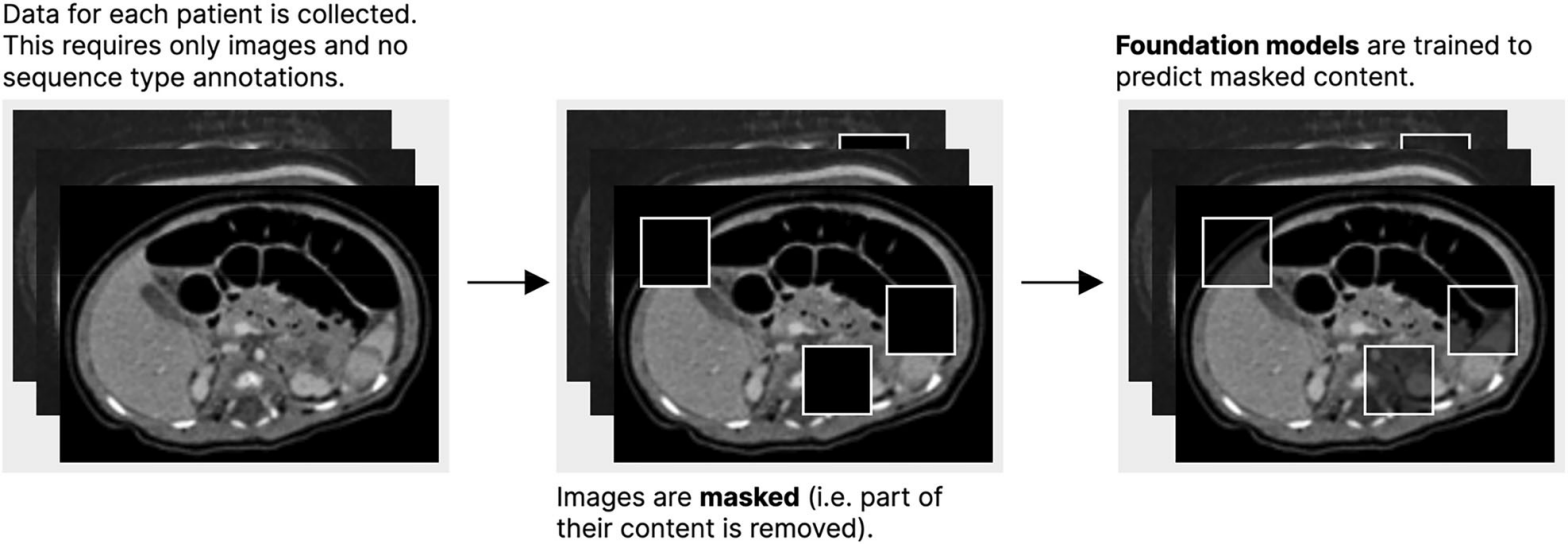
Pre-training with masked auto-encoders. Small parts (also known as patches) are removed from each image. A model will then learn how to predict the image content in each missing patch, thus creating a model capable of capturing rich contextual information without having access to the entire image

**Fig. 4:**
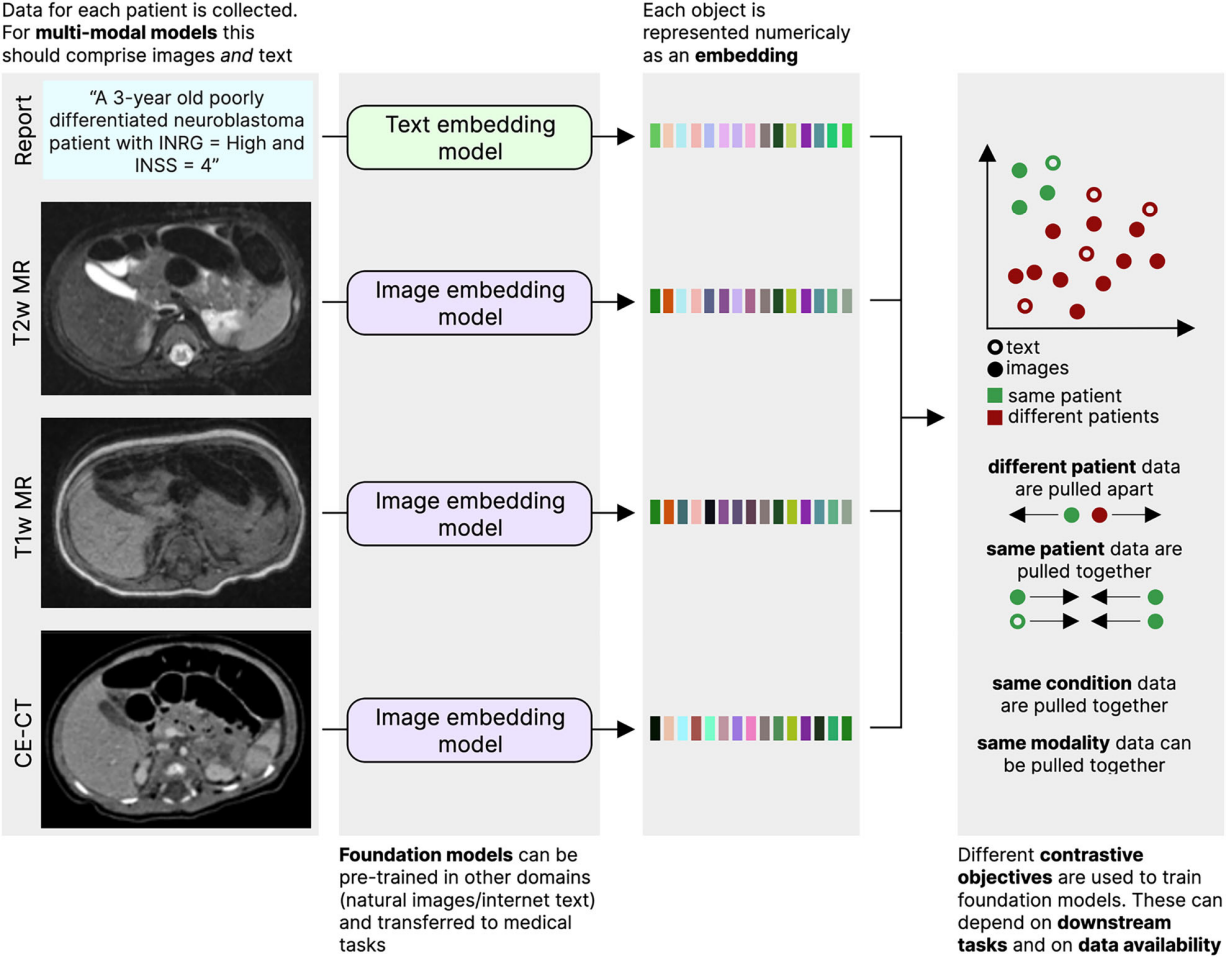
Pre-training with contrastive learning. Input images may have paired text data (i.e., reports), but this is not required. Each image or report is converted to a numerical embedding, and these numerical vectors are approximated if they have common characteristics (same condition, patient or modality) and pulled apart if they have something conflicting (different patients or conditions, for example). In the end, embeddings are capable of characterizing both images and reports in a way that is semantically meaningful, and their embeddings cluster in this high-dimensional space

In the field of radiology, pre-training with no annotations has been performed extensively. These approaches include report or caption generation for medical images (allowing models to learn which parts of the image are relevant for radiologists; Fig. 2) [58, 59], masked autoencoders, (parts of the image are removed and a model is trained to predict the missing parts; Fig. 3) [60], and contrastive learning (a model learns how to numerically characterize an image consistently despite alterations to its content [61–64]; Fig. 4). Other approaches make use of annotations to derive foundation models. MedSAM [65], for instance, is a well-performing model for generic assisted medical image segmentation. Based on the Segment Anything Model (SAM) [66], MedSAM was trained to generate segmentation masks for different anatomically relevant regions using bounding boxes (rectangles or cubes that enclose a given anatomical region and which can thus be semi-automatically segmented with a high level of accuracy).

A particular advantage of vision foundation models is that they typically perform well when applied to external datasets [58, 65, 67]. They also require fewer annotated data to achieve better performance when compared with models trained using supervised learning alone [61, 64, 67]. For text-based tasks, some pre-trained LLM-based applications in impression and finding generation from radiology reports have shown potential [68–70]. Particularly, through few-shot learning—where image classifications are performed using a pre-trained model and only a few examples—generic foundation models can be adapted to medical image classification tasks [71]. Zero-shot learning—where no examples are provided in the training data—has also been shown to be more data-efficient (i.e., to require fewer training examples) when compared with supervised alternatives [72, 73].

However, as is the case with any AI tool, the performance of foundation models is bounded by the errors, limitations and biases of both their human creators and the data on which they have been trained. Known limitations of foundation models include:

1. **Lack of transparency and explainability:** due to the extremely large number of parameters and probabilistic associations found in the training data that are difficult to interpret, models become harder to explain.
2. **Confabulations/hallucinations:** model outputs may appear to be realistic (i.e., follow the typical structure of real data) but be non-factual. This includes the generation of false information [74, 75] and leads to high rates of inaccuracies in several different applications [76].
3. **Catastrophic forgetting** [77]**:** when models are fine-tuned on small datasets, their performance or alignment with human values and safety principles may drop when tested on samples or datasets where the model used to perform well [78–80]. In other words, trying to adapt foundation models to specific tasks may lead to them losing performance on tasks where they previously performed well.
4. **Bias:** foundation models may be biased against specific categories or characteristics. For instance, foundation chest X-ray models can exhibit biases in terms of gender and race, making their generic application problematic; indicatively, performance decreased when identifying chest radiographs with no findings and with pleural effusion for female and black individuals, respectively [81]. A systematic study of foundation models for medical images showed that sex and race biases are pervasive across foundation model pre-training approaches, despite the use of large amounts of data, and that increasing the amount of pre-training data or fine-tuning on balanced datasets leads only to partial mitigation of biases [82]. As an illustrative example, we refer to RETFound—a foundation model for retinal images [53]. While remarkable in its performance, a later analysis on whether RETFound, trained on a diverse Western population, could generalize to an Asian population showed that this foundation model did not provide an advantage compared to foundation models trained on natural image (i.e., non-medical imaging) data [83].

Additionally, while similar in many ways, key differences exist between natural and clinical images, which make training clinical image foundation models more difficult:

1. Dataset size: ImageNet, a widely available natural image dataset, contains over 14 million images [84], while private datasets like those owned by Meta reach into the billions [85]. In contrast, the largest chest radiograph dataset—MIMIC-CXR [86]—has approximately 350,000 annotated X-rays. Efforts have been made to bridge this gap. To close this gap, datasets like SA-Med2D-20M have compiled 20 million masks across 4.6 million 2D medical images (58.4% from clinical imaging) [87–89]. These datasets are built by pooling publicly available sources—over 100 for SA-Med2D-20M and IMed-361M, and 20 for UMIE. Despite these advances, acquiring and annotating new clinical image data remains the primary challenge.
2. Dimensionality: As noted, large datasets are typically achieved by treating clinical images as 2D. However, many clinical images are inherently 3D, and this dimensionality carries crucial contextual information essential for training clinical imaging foundation models. Some large 3D datasets exist: CT-RATE includes over 25,000 CT studies [90], and the UK Biobank is collecting cardiac, abdominal, and brain MRIs from up to 100,000 individuals [91]. For segmentation, datasets from BraTS challenges offer a few thousand annotated brain tumor studies [92]. The datasets used to train TotalSegmentator (over 1200 CT studies) and TotalSegmentator MRI (over 500 CT and 600 MRI studies) used high-quality annotations for over 100 anatomical structures [93, 94].
3. Signal distribution: unlike natural images, which vary widely (e.g., different animals are easily distinguishable), medical images tend to be highly standardized and similar across individuals due to years of clinical protocol development. Moreover, diagnostically relevant features—hepatocellular carcinoma in abdominal CT, prostate lesions in multiparametric MRI, or intracranial hemorrhages in head CT—typically occupy only a small portion of each image. Although not yet well studied, this subtlety and limited variability may complicate model optimization.
4. Inter-rater variability in annotation and annotation quality: different people may annotate objects and images differently. While this is not particularly discussed in natural images, inter-rater variability in annotations is of paramount importance in clinical imaging. Shwartzman and others recently showed that training a model on annotations from a single individual amplified the inter-rater variability of model outputs [95] in brain MRI. Similarly, decreasing the inter-rater variability in cell segmentation in histopathology led to improved performance despite smaller datasets [96].

Finally, when researchers assessed LLMs and, more recently, vision language models (VLMs) for their performance in medical licensing examinations, the performance was quite remarkable [97–99]. This naturally created a flurry of research and publications in the field of radiology using commercial LLMs for a wide array of tasks (such as diagnosis, report summarization or impression generation). However, results were mixed [76, 100, 101]: LLMs produce biased responses in medical [102, 103] and non-medical contexts [104, 105]. Additionally, certain medical LLM applications have proven to be worse than their human expert equivalent at impression generation [106, 107] and medical evidence summarization [108].

## Foundation models as part of the future of AI in clinical practice

To answer, “How can we get models that perform well on present-day data,” foundation models may very well be the solution. However, if we ask how a robust and consistent clinical AI-supported ecosystem can thrive and practically serve patients and clinicians in years to come, foundation models are only part of the solution.

Foundation models can indeed bridge to some extent an existing gap in the generalization of machine-learning applications in the clinic by making use of medical knowledge existing in vast datasets. However, they may still be affected by some of the same limitations (outlined above), causing non-foundation models to remain largely unadopted in clinical practice. While the development of well-performing models is important, holistic validation in terms of trustworthiness and continuous learning is equally essential to ensure practical clinical utility and patient safety in an ever-evolving healthcare environment.

Modern decentralized approaches to medical data curation and federated storage, such as those applied in the Cancer Image Europe federated network, whose aim is to provide large amounts of data and federated learning/federated data processing approaches for medical research and experimentation [109], can act as groundbreaking foundations for the consistent training, validation, monitoring and continuous improvement of foundation models. Such frameworks may act as enablers for state-of-the-art AI modeling approaches, including foundation models and generative AI tools, by providing the data volume, variety, multimodality, and quality required for their extensive validation and retraining while preserving patient privacy.

Recent literature can also provide important insights into what a consistent, multi-centric, and continuous clinical model validation and training framework can look like. VAI-B, a Swedish national project focusing on the external validation of models, collects data from multiple institutions and, through careful orchestration of different models and their input/output requirements, is capable of delivering accurate estimates for the external performance of multiple models [110]. Similarly, RACOON, a German network of medical centers focusing on data collection for federated learning [111], shows promising results in coordinating between model training in institutions with good computational resources and model validation in institutions with fewer computational resources. Such an arrangement ensures that every institution can be involved in model training and validation. Similar approaches of continuous data collection and decentralized model validation and/or training could be deployed to not only train but also validate promising foundation models.

Integrating human-in-the-loop approaches and learning from clinical expert-user’s feedback [52, 112] to continually improve AI tools and render them dynamically adaptable and generalizable to ever-changing clinical practice operational conditions is especially important when using foundation models to address specific tasks [113]. Of equal importance is building “self-awareness” in foundation models by integrating awareness of the model’s limitations and uncertainties, e.g., by deploying uncertainty estimation techniques [114, 115] and providing mechanisms that enable AI to ask for human intervention or feedback when uncertainty is high, e.g., through clarification questions [116].

Holistic AI trustworthiness frameworks such as FUTURE-AI—which provides recommendations and guidelines for adherence to 6 main principles of trustworthiness: fairness, universality, traceability, usability, robustness and explainability [30]—can also act as critical guidance in the development, validation and deployment of foundation models that are trustworthy and have higher chances to be used in clinical practice. An important obstacle to trustworthiness lies in LLMs developed by large companies—such as ChatGPT by OpenAI or Gemini by Google. This is because data documentation for the pre-training of these models is limited or altogether non-existent, while datasets can be proprietary and inscrutable when models are further optimized using reinforcement learning with human feedback [52] or similar approaches. This is in direct confrontation with the traceability principle of the FUTURE-AI framework, suggesting that the whole lifecycle of the model—including its training process and data—be adequately documented and monitored. It is also in direct confrontation with the European Union’s AI Act, which requires high-risk AI systems (including clinical AI models) to be fully transparent about their training process and data [117].

Addressing the lack of transparency in proprietary models may involve the use of open LLMs, which openly document data—LLaMa models started by accurately documenting data sources which were used during training [118], while projects such as Pythia go the extra mile by providing the code necessary for full replication [119]. Technical developments in model explainability can also render model output easier to understand and increase the trust in their output—as highlighted in a recent review, LLM explainability can be achieved at several different levels, some of which mimic easily understandable explanations [120].

## Conclusion

Here, we outline issues surrounding modern approaches to clinical machine-learning models using medical imaging, and consider how the development of a larger landscape of foundation models could partially address them. However, foundation models on their own are not sufficient to solve inherent biases or subpar generalization, particularly if there is a tendency to assume that these are entirely solvable without appropriate computational and data infrastructure. We thus recommend that efforts should focus on building diverse, well-documented datasets that involve clinical experts while enabling collaborative and decentralized training. Finally, the clinical and research communities should strive to certify that foundation models are transparent, clinically relevant, and broadly applicable. We expand on these efforts in Table 3.

**Table 3:** Practical recommendations regarding the future development of foundation models for clinical imaging, and which problems they address

Problem to be addressed	Recommendation	Expected outcome
Demographic biases	Efforts should be directed not only toward the implementation and development of foundation models, but also toward the creation of an appropriate and demographically balanced data and validation infrastructure.	Better performance across different population categories, including race, nationality, gender, and age. Additionally, better performance across different acquisition conditions and centers.
Domain knowledge Adaptation to novel and changing scenarios	Foundation model development (continuous and otherwise) for radiology should prioritize the active involvement of experts (i.e., radiologists and clinicians) to ensure that these models remain well-adapted to clinical circumstances.	Better model performance tracking and more participative involvement of medical professionals in AI system development.
Lack of open and annotated data Excessive variability in annotations	A concerted effort in clinical image data collection—both 2D and 3D—and annotation that allows the construction of consistent and publicly available datasets, enabling a more productive ecosystem for the development of research and commercial applications. The use of data standards for annotation, such as RadLex, can assist in these efforts [134].	Open datasets with better and more consistent annotations.
Lack of data diversity Lack of stakeholder involvement	A plural and holistic involvement of institutions in either decentralized model training and/or validation is necessary, as this will guarantee data diversity, traceability and accountability.	More diverse data, which also addresses potential biases. Better preservation of patient privacy through the use of federated/decentralized methods. Reduction of the amount of resources used to train foundation models by orchestrating large consortia with common goals.
Lack of open models Performance gap between proprietary and open models	Foundation models should be developed in a way that guarantees their widespread applicability and inspectability from a technical perspective: open source software and ample data documentation can satisfy this.	Methods that are more transparent, more reproducible and easier to validate across different centers.

We posit that these recommendations can make foundation models more transparent, robust and performant while also increasing the trust from both medical professionals and patients alike.
